# Editorial

**Published:** 1847-08

**Authors:** 


### THE MEDICAL EXAMINER.

### PHILADELPHIA, AUGUST, 1847.

### NATIONAL MEDICAL CONVENTIONS.

We have received the official copy of the "Proceedings of the National Medical Conventions, held in New York, May, 1846, and in Philadelphia, May, 1847." An account of the Convention which assembled in New York was published in the Medical Examiner last year, and a considerable portion of the proceedings of that held in Philadelphia has likewise been laid before our readers, and the remainder will appear as we can afford space. In our Record department of the present number will be found the "Report" of the committee on "a uniform and elevated standard of requirements for the degree of M. D." The *report* is plainly written, and manifests throughout good temper and honorable purpose; but its reasonings, to our mind, are not always conclusive; not because of any lack of inductive power on the part of its authors, but from the want of a proper attention to some of the facts which are involved in the questions discussed.

The Committee, it will be seen, institute a comparison between the requirements for medical honors in the United States and those demanded in the oldest countries and most absolute governments of Europe, where the precepts of the Colleges are enforced by the strong arm of the law, and where every licensed practitioner is protected in the exercise of his profession; and where, too, in the strongest instances cited, the educational expenses of the student are borne by the government.

"We are free to confess," say the Committee, "that within the last twenty or five and twenty years, medicine has been better taught among us than at any former period."

This is certainly true; and we are glad to have the testimony of the Committee to the fact, for it would seem to be the opinion of some enthusiasts on the subject of "reform in medicine," that every thing is growing worse, and that medical instruction, as well as every other thing medical, is absolutely approaching the lowest point of degradation.

We are probably correct when we say that a majority of the Convention which adopted the declaration we have cited, as well as several of the committee from whence it emanated, received *their* medical education under circumstances and at a time when, according to the *report*, medicine was not so well taught among us as at present; and yet we do not believe that any one of these respectable gentle. men thinks, or would have others to think, that *his* education was so defective as to disqualify him for the proper performance of the duties of a physician; and if not, with what justice can such a con. clusion be predicated of those who now enjoy the *greater* advantages admitted by the report!

Again—"It is unquestioned," says the *report*, "that the facilities for education have been augmented, not only in the public schools of the country, but private enterprise has exerted no slight influence in the attainment of this end."

That "the facilities for education have been augmented," and, in the larger medical schools, at least, are every year "augmented," is a fact familiar to all who have enjoyed opportunities for observing. This, one would think, ought to satisfy well wishers of our profession that the present system—a system which, it is confessed, leads to regular improvement—should not be hastily or inconsiderately changed. The "private enterprise" alluded to, we understand to mean, the numerous associations and individuals engaged in lecturing on the various branches of medicine and collateral subjects; and we are happy to agree with the report in the estimation of their utility. These private teachers are found in all our large cities where Medical Colleges exist, and, by occupying the summer months, in effect extend the term of instruction, which students may enjoy, to eight months or more in the year. Not only is "the term" extended in this way, but students have the opportunity of hearing the views of different teachers on the same subjects, and are thus led to compare and examine for themselves; and what is also important, the perpetual tread of these aspirants for professional honors upon the heels of those preceding them, prevents the possibility of the latter falling back, or even standing still. But fill up a large part of the year with college exercises, and this "private enterprise" to which the report accords, and justly accords, so much good, will be crowded out of existence—a result which certainly none but lazy and incompetent professors ought to desire.

We are glad to perceive that the *report* attaches much importance to the efforts of "private preceptors" in elevating the standard of our profession—a subject upon which we shall probably offer some remarks in a future number.

After an enumeration of the branches proposed to be taught in the schools—which are precisely those now taught in our principal colleges—the *report* argues: "With a lengthened period for teaching, a double advantage will be gained; a wider extent of information may be imparted to a student, while his time will be occupied with fewer lectures during the day." If "a wider extent of information" can be imparted in fewer lectures, most teachers will be glad to learn how; but if it require that *the number of lectures shall be increased* to attain this end, and fewer are to be given each day, then, according to our computation, it will require a very considerable extension of the term to accomplish the object. Among the resolutions passed by the late Convention, was one requiring that not less than *one hundred* lectures should be given on any one of the branches; but, on discovering that this would nearly *double* the number of lectures now given on certain of the branches, while the scheme of the Convention only contemplated *one-third* additional time, it was rescinded on the very next day—shewing that, however desirable the change, it was then deemed neither practicable nor expedient.

The first *resolution* reported by the committee, and adopted by the

Convention, recommends "to all the Colleges to extend the period employed in lecturing from four, to six months." Believing the proposition to be one of doubtful expediency, and that no conference among the Colleges in reference to the matter had occurred or was likely to occur, at least before their next sessions, we have felt some interest to know what course would be generally pursued on the subject. We have now before us the advertisements of most of them, from Maine to Louisiana, and from the Lakes to the Atlantic, and with a single exception, we have not discovered any change in their former course, that looks like conforming to this recommendation. In the excepted case, the only departure from the course hitherto pursued, as far as we are advised, consists in the annunciation that the lectures "will commence on Monday, October 18th," instead of the first Monday of November, and extend to the end instead of terminating at the middle of March. We have, we confess, been surprised at the almost unanimous concurrence in the action of the Colleges in this matter—the more remarkable because it is without concert or any conference whatever. That so many institutions, widely separated, placed under diverse circumstances and controlled by different councils, should arrive at the same conclusion, in opposition to the recommendation, and the *threat* contained in one of the resolutions, of the Convention, is indeed very remarkable, and shows clearly enough what those who have experience in the matter think on the subject,—that it is environed with greater difficulties than some have contemplated.

The tenth and last *resolution*—"That it be considered the duty of Preceptors, to advise their students to attend only such institutions as shall rigidly adhere to the recommendations" of the Convention, seems to be unheeded by them all. We do not know of a single Medical College in the United States that rigidly adheres to all of the recommendations, or proposes to do so.

The whole subject, in fact, of education, is one of very great difficulty,—particularly where masses are concerned, the individuals of which are so unequally circumstanced as are young men spread over an extensive country, with different habits, means, objects and indications, such as we find engaged in the pursuit of medicine as a profession. On another page we have copied some remarks on this subject, by Dr. Latham, which are deserving of attention both from the learning and eminence of their author and the sound common-sense views they express.

### CATALOGUES OF MEDICAL COLLEGES.

We have received the catalogues and annual circulars of the following institutions, viz.:

*University of Louisville.*—The last Legislature of Kentucky granted a charter for a University, under which the late Medical Institute of Louisville has become the Medical, and the suspended Louisville College is to become the Academical Department; a Law Department being also added. Number of the medical class of the last session, 348.

*Medical Institution of Geneva College.*—Number of Graduates at the last commencement, 43.

*Medical Department of Transylvania University.*—Class 205; Graduates, 68.

*Willoughby Medical College.*—This institution has been removed to Columbus, Ohio; number of the last class, 90.

*Baltimore College of Dental Surgery.*—This institution continues its useful career as heretofore, under the same Faculty and scheme of instruction.

